# Neuroadaptability and Habit: Modern Medicine and Ayurveda

**DOI:** 10.3390/medicina57020090

**Published:** 2021-01-21

**Authors:** Robert Keith Wallace, Ted Wallace

**Affiliations:** Department of Physiology and Health, Maharishi International University, Fairfield, IA 52556, USA

**Keywords:** neuroadaptive, stress, P4 medicine, Transcendental Meditation, Ayurveda, gut bacteria, diet, lifestyle, disease, prevention, integrative medicine

## Abstract

In our increasingly stressed world, especially with the COVID-19 pandemic, the activation of the threat network in everyday situations can adversely affect our mental and physical health. Neurophysiological response to these threats/challenges depends on the type of challenge and the individual’s neuroadaptability. Neuroadaptability is defined as the ability of the nervous system to alter responsiveness over time to reoccurring stimuli. Neuroadaptability differs from neuroplasticity, which is more inclusive and refers to the ability of the nervous system to change and learn from any experience. We examine neuroadaptability and how it affects health from the perspective of modern medicine and Ayurveda.

## 1. Introduction

The human body has a remarkable ability to resist external change and maintain internal order and coherence. The concept of homeostasis is at the foundation of physiology and medicine. Neuroadaptability provides a measure of how effectively these homeostatic mechanisms are working at the neural level.

Numerous studies suggest that the stress network in the brain, involving areas such as the paraventricular nucleus, amygdala, and hippocampus, under certain circumstances, plays a significant role in mental and physical disorders as a result of poor neuroadaptive responses and the long-term modification of gene expression, receptors, and neural pathways. Chronic high resting levels of the stress hormone cortisol is associated with the suppression of the immune system and the destruction of cells in the hippocampus. Stress has been identified to play a role in the pathogenesis of a number of disorders such as high blood pressure, cardiovascular disease, anxiety, and depression [1,2,3,4,5,6].

Neuroadaptability covers a wide spectrum of responses to challenge and can produce positive and negative results in terms of health. At one end of the spectrum, we find the repeated inappropriate activation of the full fight or flight response, creating a state of chronic stress, while at the other end of the spectrum is the ability to respond appropriately to stress, and to recover quickly, returning to an ideal state of integrated function. 

What factors affect neuroadaptability? Stress and addiction are the main models for “poor” or ineffective neuroadaptive behavior and habit. Increased stress is affecting everything we do and putting greater demands on our neuroadaptability. In various types of addiction, genetics, environmental, and social influences play a significant role. There is extensive research on the complex transcriptional and gene expression mechanisms that underly this type of neuroadaptive behavior. Excessive stress, especially in childhood, is also a major factor in the development of addiction and other negative habits [1,4,6]. 

In addition to psychological stress, we must also take into account other lifestyle influences. Recent research on the gut–brain axis reveals the dynamic communication which takes place between the nervous system, endocrine system, immune system, enteric nervous system, and gut microbiome. It is now clear that a disruption to the microbiome can in turn affect the nervous system and general state of health [7,8,9,10,11]. 

To improve neuroadaptability, we must learn to manage various types of challenges. How, for example, should we handle the increasing number of toxins in the environment? Each challenge, each stress, puts an increasing load on the body from the level of DNA to the nervous system. McEwen defines allostatic load as the effect of chronic exposure to elevated or fluctuating endocrine or neural responses as a result of specific repeated stressful challenges [1]. 

Integrative medicine deals with stress primarily by recommending a preventative lifestyle approach, recognizing that to maintain good health we must consider all aspects of our life including diet, sleep, exercise, and stress management. One interesting new area of medicine is P4 medicine, which emphasizes four Ps: predictive, preventative, personalized, and participatory [12,13,14,15]. These same factors have been an integral part of traditional systems of medicine, including Ayurveda from the Vedic tradition of India [16]. 

In this article, we highlight two key approaches to improve effective neuroadaptability. The first is meditation and the second is Ayurveda. This is slightly arbitrary since meditation is, in fact, an integral part of Ayurveda but we will consider them separately for the purposes of this article. Many forms of meditation are practiced around the world. We will focus on an automatic self-transcending technique, which comes from the Vedic tradition, specifically Transcendental Meditation (TM), because of the large body of research that demonstrates its ability to improve neuradaptability and mental and physical health. We will then consider Ayurveda and discuss how adopting more individualized lifestyle habits can also be used to improve specific areas of neuroadaptability [17,18,19].

## 2. Transcendental Meditation

It is important to clarify that different meditation techniques produce different physiological changes. One review paper proposed three types of meditation practices, classified according to measures of EEG frequency, power, and coherence, as well as the use of brain imaging techniques. Further, it is important to note that not all approaches of meditation have the same effect at preventing or reversing the effects of stress [20,21].

Early studies on subjects practicing Transcendental Meditation showed increased skin resistance, decreased breath rate, oxygen consumption, and plasma lactate, and EEG alpha waves in the frontal areas of the brain during the practice [22,23,24]. The early studies also showed decreased cortisol levels during TM and lower levels of cortisol both during the day and at night in a TM group as compared to controls [25,26]. These initial findings have been expanded and extended by a number of researchers who have reported positive effects on autonomic, biochemical and brain activity measures [27,28,29,30,31,32,33,34,35,36,37,38]. 

## 3. Autonomic and Other Measures in Response to Stress 

How does the repeated experience or regular habit of practicing Transcendental Meditation affect neuroadaptability? In an initial study, it was shown that TM meditators had fewer spontaneous skin resistance responses outside of meditation than non-meditating controls. The study also measured evoked skin resistance responses in meditators and non-meditators who were presented with unpleasant tones at regular intervals. The TM meditators habituated significantly faster than the non-meditators. Further, meditators produced fewer multiple fluctuations in skin resistance during the recovery cycle, suggesting a more stable reaction to stress [39]. 

These results were extended in another study in which researchers investigated the response pattern to stressful stimuli of TM meditators as compared to a relaxation control group using continuous measures of heart rate and phasic skin conductance, as well as self-report in personality scales. The groups either meditated or rested after they viewed a film of workshop accidents. The meditators’ heart rates increased more than the non-meditators’ in anticipation of the accidents but recovered more quickly than the non-meditators’ after the films. Phasic skin conductance responses also increased more in meditators with anticipation of the accident scenes, but again habituated more quickly. TM practitioners also reported experiencing less subjective anxiety [40]. 

In a prospective, random assignment study, changes in baseline levels and acute responses to laboratory stressors were measured in practitioners of the TM technique and compared to a stress education control group. Cortisol, growth hormone, thyroid-stimulating hormone and testosterone were measured before and after 4 months of either TM or stress education. The basal cortisol level and average cortisol across the stress session decreased for the TM group. However, the cortisol responsiveness to stressors increased compared to controls. The baselines and stress responsiveness for TSH and GH were different for the TM group as compared to controls, and there were differences in the testosterone baseline [41].

One study examined the impact of the TM program on cardiovascular reactivity in 35 adolescents with high normal blood pressure. The subjects were randomly assigned to either the TM or the health education control group. There were two stressors: a simulated car driving stressor and an interpersonal social stressor interview. After two months of TM practice, meditating subjects were able to drive at a higher maximum speed on a driving simulator with less elevation of blood pressure during driving as compared to the control group [42]. 

A study using fMRI showed lower brain activation in thalamus and total brain areas in response to a temperature stress in long-term TM participants as compared to healthy controls. There was also a decrease in pain sensitivity in the TM group. After the controls learned and practiced the TM technique for 5 months, they showed results similar to the long-term TM participants [43]. 

A randomized controlled trial studied the effects of TM practice in 50 college students using the following measures: Brain Integration Scale scores, which measures broadband frontal coherence, power ratios, and preparatory brain responses; measures of electrodermal habituation to loud tones; sleepiness; heart rate; respiratory sinus arrhythmia; and P300 latencies. After pretest, subjects were randomly assigned to learn TM immediately or learn after the 10 week post-test. There were no significant pretest differences in the groups. The results showed significant differences between the TM subjects and the controls (delayed-learning TM group) for the Brain Integration Scale scores, sleepiness, and habituation rates [44]. 

A randomized controlled study of 27 college students found that four weeks of TM practice significantly reduced the level of cortisol when waking up from a night’s sleep as compared to controls [45]. 

These studies together suggest that TM produces a more stable internal resting state of autonomic and biochemical activity, which provides the basis for a more effective ability to adapt to environmental stresses.

## 4. Clinical Studies and Aging

Research studies have documented the positive effects of TM on health. The most well-studied area is on the effects of TM on reducing blood pressure and improving cardiovascular disease. Many of these studies were carried out in collaboration with major medical schools and have been performed with the most rigorous and well-controlled designs. Several review papers show that TM significantly reduced both systolic and diastolic blood pressure, while none of the other treatments, which ranged from simple biofeedback, relaxation-assisted biofeedback and progressive muscle relaxation, to stress management training, had a significant effect on blood pressure. These reviews also include studies which showed that TM affects other risk factors such as lowering cholesterol levels; reducing smoking; improving fasting blood glucose and insulin levels in patients with metabolic syndrome; reversing arteriosclerosis; and improving functional capacity and quality of life of patients with congestive heart failure [46,47,48]. 

One particular study was a randomized controlled trial of 201 African American men and women with coronary heart disease at the Medical College of Wisconsin in Milwaukee. Measured over five years, the middle-aged and elderly African Americans had an average age of 58. They were randomly assigned to either a health education group or to a TM group. The primary measures were mortality, myocardial infarction, and stroke. After an average follow up of 5.4 years, there was a 48% risk reduction for these measures in the TM group as compared to controls [49]. 

A number of other studies have shown that TM reduced overall medical utilization and expenses for many different diseases, particularly for cardiovascular and neurological disorders [50,51,52,53]. One randomized controlled trial of 130 women showed that Transcendental Meditation improved quality of life in older breast cancer patients [54]. 

A recent well-controlled study of veterans with Post-traumatic stress disorder (PTSD) compared the practice of TM with prolonged exposure therapy and PTSD health education. The researchers found a significant decrease in the severity of PTSD symptoms in veterans who learned TM [55]. 

In the area of aging, a reduction in biological aging was shown in one study; another showed improvement in cognitive and behavioral flexibility, health and longevity in elderly individuals who learned TM as compared to controls practicing mindfulness and relaxation [56,57]. An eight-year follow-up study of older blood pressure patients found that those who were randomly assigned to the Transcendental Meditation technique had significantly lower rates of mortality than controls [58]. 

One preliminary study examined the effects of TM on gene expression and found over 70 genes to be changed in practitioners of TM as compared to control groups, which may help explain the many health benefits [59]. 

## 5. Psychological, Substance Abuse, and Business Studies

Two studies using meta-analysis have shown decreased anxiety in TM subjects as compared to control subjects practicing a variety of different relaxation and mindfulness techniques [60,61]. A large number of other psychological studies have shown many different improvements such as reduced depression, increased intelligence, and increased self-actualization. In addition, studies have shown reduced cigarette smoking, alcohol use, and substance abuse, lower prison recidivism, increased productivity, and improved work and personal relationships in business [62,63,64]. These studies include a wide variety of conditions, which include both reacting to and coping with different types and amounts of stress.

## 6. Summary TM Research

The overall conclusion is that the TM technique appears to create a state of high internal stability and coherence, which results in more effective neuroadaptability and better physical and mental health. In examining the evolution of life, physiologists recognize that greater internal balance is achieved through the development of more refined homeostatic mechanisms. This enables higher organisms to be more adaptable and therefore freer from the deleterious effects of living in a changing environment [65].

**Figure 1:**
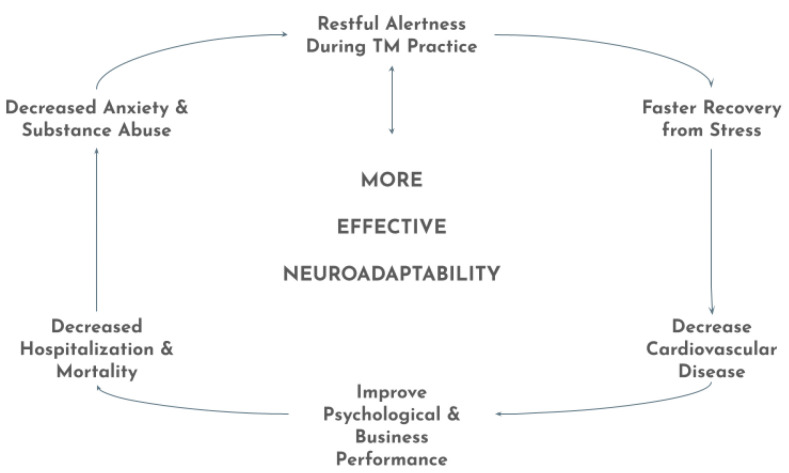
Effective Neuroadaptability with Regular Practice of Transcendental Meditation Caption.

What future studies can be performed? A more complete analysis of how changes in gene expression specifically affect physiological functions as a result of TM might give us greater insight into the molecular mechanism of more effective neuroadaptability. Is it possible to develop optimal functioning of human homeostatic processes? Further studies on TM may give us an understanding of how this one simple habit can simultaneously improve so many areas of our health, including decreasing certain negative habits such as addiction to cigarettes, alcohol, and drugs (see Figure 1) [63].

Another complementary approach for increasing neuroadaptability is Ayurveda.

## 7. Ayurveda

Ayurveda means knowledge or science of life. One of the most important concepts in Ayurveda, according to the Charaka Samhita, is an understanding of different psycho-physiological constitutions, called Prakriti [16]. 

Each individual can be broadly categorized into one of seven different mind/body types or Prakriti. These types are based on the balance or proportion of three fundamental physiological principles, referred to as the three doshas—Vata, Pitta and Kapha. There are a number of studies which show that there is correlation between Prakriti and gene expression, thus helping to provide a modern scientific understanding of Ayurveda [18]. Proper health depends upon keeping the doshas in balance. Aggravation or imbalance leads to disorder and disease. 

The concept of stress is well described in Ayurveda. Each Prakriti reacts differently to stress. A Vata Prakriti, for example, reacts with anxiety, while a Pitta reacts with anger. The prognosis for and susceptibility to different diseases also vary depending upon the predominance of these three doshas in the individual. Many stress-related conditions are related to a Vata aggravation or disorder. Thus, establishment of the individual’s body type is crucial for diagnosis as well as treatment [16]. Ayurveda includes a wide variety of stress management techniques. Some are specific to each Prakriti while others, in particular Transcendental Meditation, can be used by all individuals.

Ayurveda places a huge emphasis on diet and digestion and provides an elaborate nutritional and dietary program. The main preventative approach of Ayurveda is the recommendation of individualized lifestyle habits for daily and seasonal routines. The recent findings of the role of the gut microbiome in health help provide a scientific understanding of the importance of an individualized diet in Ayurveda. It is proposed that the personalized lifestyle habits of Ayurveda also utilize epigenetic mechanism to create greater balance and adaptability in the physiology [17,19].

## 8. Conclusions

Transcendental Meditation is a mental technique, which improves overall neuroadaptability and health. Ayurveda recommends many positive individualized habits, which improve neuroadaptability in specific areas of life, and, as we mentioned, it includes the four principles of P4 medicine: predictive, preventative, personalized, and participatory. Transcendental Meditation and Ayurveda could be used along with other techniques such as coaching to improve long-term psychological and physiological balance, modify molecular and neurophysiological mechanisms, and improve neuroadaptability and performance in life and in business [65,66].

We suggest that by combining Transcendental Meditation and Ayurveda with the latest knowledge of modern medicine, we can create a more complete and effective system to prevent disease and improve physical and mental health [67].
